# Regulatory effects of the JAK3/STAT1 pathway on the release of secreted phospholipase A_2_-IIA in microvascular endothelial cells of the injured brain

**DOI:** 10.1186/1742-2094-9-170

**Published:** 2012-07-12

**Authors:** Guansong Wang, Pin Qian, Zhi Xu, Jiqiang Zhang, Yaoli Wang, Saiyu Cheng, Wenqin Cai, Guisheng Qian, Changzheng Wang, Mark A DeCoster

**Affiliations:** 1 Neuronscience Program, Institute of Respiratory Diseases in Xinqiao Hospital, Chongqing, 400037, P. R. China; 2 Department of Neurobiology, The Third Military Medical University, Chongqing, 400037, P. R. China; 3 Biomedical Engineering and Institute for Micro Manufacturing, Louisiana Tech University, Ruston, LA, 71272, USA

**Keywords:** Secreted phospholipase A_2_-IIA, Brain microvascular endothelial cells, Permeability, Lipopolysaccharide, Nitric oxide, Inducible NO synthase, JAK3, STAT1

## Abstract

**Background:**

Secreted phospholipase A_2_-IIA (sPLA_2_-IIA) is an inducible enzyme released under several inflammatory conditions. It has been shown that sPLA_2_-IIA is released from rat brain astrocytes after inflammatory stimulus, and lipopolysaccharide (LPS) and nitric oxide (NO) have been implicated in regulation of this release. Here, brain microvascular endothelial cells (BMVECs) were treated with LPS to uncover whether sPLA_2_-IIA was released, whether nitric oxide regulated this release, and any related signal mechanisms.

**Methods:**

Supernatants were collected from primary cultures of BMVECs. The release of sPLA_2_-IIA, and the expression of inducible nitric oxide synthase (iNOS), phospho-JAK3, phospho-STAT1, total JAK3 and STAT1, β-actin, and bovine serum albumin (BSA) were analyzed by Western blot or ELISA. NO production was calculated by the Griess reaction. sPLA_2_ enzyme activity was measured with a fluorometric assay. Specific inhibitors of NO (L-NAME and aminoguanidine, AG), JAK3 (WHI-P154,WHI), STAT1 (fludarabine, Flu), and STAT1 siRNA were used to determine the involvement of these molecules in the LPS-induced release of sPLA_2_-IIA from BMVECs. Nuclear STAT1 activation was tested with the EMSA method. The monolayer permeability of BMVECs was measured with a diffusion assay using biotinylated BSA.

**Results:**

Treatment of BMVECs with LPS increased the release of sPLA_2_-IIA and nitrite into the cell culture medium up to 24 h. Pretreatment with an NO donor, sodium nitroprusside, decreased LPS-induced sPLA_2_-IIA release and sPLA_2_ enzyme activity, and enhanced the expression of iNOS and nitrite generation after LPS treatment. Pretreatment with L-NAME, AG, WHI-P154, or Flu notably reduced the expression of iNOS and nitrite, but increased sPLA_2_-IIA protein levels and sPLA_2_ enzyme activity. In addition, pretreatment of the cells with STAT1 siRNA inhibited the phosphorylation of STAT1, iNOS expression, and nitrite production, and enhanced the release of sPLA_2_-IIA. Pretreatment with the specific inhibitors of NOS, JAK2, and STAT3 decreased the permeability of BMVECs. In contrast, inhibition of sPLA_2_-IIA release increased cell permeability. These results suggest that sPLA_2_-IIA expression is regulated by the NO-JAK3-STAT1 pathway. Importantly, sPLA_2_-IIA augmentation could protect the LPS-induced permeability of BMVECs.

**Conclusion:**

Our results demonstrate the important action of sPLA_2_-IIA in the permeability of microvascular endothelial cells during brain inflammatory events. The sPLA_2_ and NO pathways can be potential targets for the management of brain MVEC injuries and related inflammation.

## Background

Brain endothelium barrier dysfunction is an important pathological process in traumatic brain injury and cerebral inflammatory disease. Brain microvascular endothelial cells (BMVECs) are the main components of the blood-–brain barrier (BBB), which performs many important functions in the nervous system. The BBB forms an active interface between the blood and brain tissue, and maintains homeostasis in the nervous system. Infections are often associated with systemic symptoms and can partly compromise the functional integrity of the BBB. The lipopolysaccharide (LPS) found in Gram-negative bacterial cell walls can take part in the activation of transcription factors in many types of cells and inflammatory diseases. LPS treatment is often used in models of cell injury or animal infection including models of infection of cerebral cells and tissues [1].

Secreted phospholipases A_2_ (sPLA_2_s) belong to a superfamily of PLA_2_ enzymes that hydrolyze the sn-2 ester of glycerophospholipids resulting in the generation of lysophospholipids and the release of fatty acids such as arachidonic acid [2,3]. Included in this superfamily are the higher molecular weight (85 kDa) Ca^2+^-sensitive cytosolic PLA_2_s (cPLA_2_s) and the calcium-insensitive PLA_2_s (iPLA_2_s), which are found inside the cell. In contrast, sPLA_2_s, including IIA and V types, are lower in molecular weight and can act in a transcellular fashion after they are secreted. sPLA_2_ is an important transcellular mediator in inflammation, as indicated by the detectably increased extracellular sPLA_2_ levels observed in, for example, atherosclerosis [4-6], acute respiratory distress syndrome (ARDS) [7], inflammatory disease [8,9], autoimmune disease [10], and allergic disorders [11]. In the brain, sPLA_2_ enzyme activity has been shown to increase after infusion with LPS [12]. sPLA_2_ mRNA and protein levels increase after ischemia [13]. The molecular basis for these cellular effects has not been established.

Glial cells, an important part of the brain endothelium barrier, respond to inflammatory stimuli, such as lipopolysaccharide (LPS), by producing more nitric oxide (NO) and releasing sPLA_2_[14]. We have shown that NO may regulate the LPS-stimulated release of sPLA_2_ type IIA (sPLA_2_-IIA) from astrocytes [15]. In the vascular endothelium of the peripheral blood vessels, sPLA_2_ has been shown to be released after stimulation with interleukin 1β [16]. Some reports have shown cross-talk between sPLA_2_-IIA and inducible NOS in some activated cells, including renal mesangial cells [17]. In addition, many studies have revealed that in vascular endothelial cells, NO expression and NOS induction are regulated by the p38MAPK, ERK1/2, JAKs, and STATs signal pathways [18,19]. For example, thalidomide has been reported to inhibit IFN-γ-induced iNOS expression and NO production by impairing STAT1 phosphorylation [20].

However, the release of sPLA_2_ from brain endothelial cells has not previously been shown. Whether sPLA_2_ expression in the MVECs of an injured brain is regulated by iNOS and JAKs/STATs remains unclear. Considering the importance of the brain endothelium in stroke and the inflammatory response after stroke, the goal of the present study was to determine the ability of BMVECs to release sPLA_2_ after inflammatory stimulus with LPS. To investigate the action of the JAK/STAT pathway in regulation of NOS expression and sPLA_2_ secretion from BMVECs, we utilized a specific inhibitor of JAK3 and STAT1 and infected the cells with STAT1 siRNA before LPS stimulus. We used nitric oxide synthase (NOS) inhibitors and NO donors to determine whether JAK3-STAT1 or NO regulate the release of sPLA_2_ from BMVECs.

## Materials and methods

### Materials

Sprague–Dawley rats were obtained from Chongqing City Laboratory Animal Center, Chongqing, China. Neuronal culture media, F-10 Nutrient Mixture medium, trypsin, and fetal bovine serum (FBS) were purchased from GIBCO, Invitrogen (Carlsbad, CA). NG-nitro-L-arginine methyl ester (L-NAME), aminoguanidine (AG), sodium nitroprusside dihydrate (SNP), endothelial cell growth supplement, DAPI, lipopolysaccharides, antibody against glial fibrillary acidic protein (GFAP), iNOS, and BSA were from Sigma-Aldrich (St. Louis, MO). Antibodies against vWF-related antigen, STAT1 siRNA [STAT1 p84/p91 shRNA (r) lentiviral particles: sc-61879-V], control shRNA lentiviral particles (sc-108080), and Polybrene® buffer (sc-134220) were purchased from Santa Cruz Biotechnology Inc. (Santa Cruz, CA). The antibody against α-SMA was purchased from Abcam plc (Cambridge, UK). The antibodies against β-actin, phospho-JAK3, phospho-STAT3, JAK3, and STAT3 were purchased from Cell Signaling Technology Inc. (Beverly, MA). WHI-P154 (inhibitor of JAK3) and fludarabine (inhibitor of STAT1) were obtained from Calbiochem Chemicals (La Jolla, CA, USA). Fluorescent substrate 1-hexadecanoyl-2-(1-pyrenedecanoyl)-sn-glycero-3-phosphoglycerol ammonium salt was purchased from Molecular Probes, Invitrogen (Eugene, OR, USA). Horseradish peroxidase-conjugated goat anti-rabbit IgG and horseradish peroxidase-conjugated goat anti-mouse IgG were obtained from Upstate Cell Signaling Solutions (Lake Placid, NY, USA). Goat-anti rabbit secondary antibody linked to fluorescein isothiocyanate (FITC) was obtained from Sigma. The sPLA_2_-IIA EIA kit was purchased from Cayman Chemical (Ann Arbor, MI, USA). Restore Plus Western Blot Stripping Buffer was from Pierce Biotechnology (Rockford, IL, USA). The instruments and software used in this study included a 3CCD camera (Bridgewater, NJ, USA), FlashBus frame grabber (Integral Technologies, Indianapolis, IN, USA), Image ProPlus software (Media Cybernetics, Silver Spring, MD, USA), and a plate reader (Bio-Tek Instruments, Winooski, VT, USA).

### Cell culture and identification

These investigations conformed to the Guide for the Care and Use of Laboratory Animals published by the US National Institutes of Health (NIH Publication No. 85–23, revised 1996) and was approved by the Ethical Committee of the Third Military Medical University of China. All rats (66) for the experiments were anesthetized with an intraperitoneal injection of 60 mg/kg body weight sodium pentobarbital, and repeated intraperitoneal injections (30 mg/kg body weight) were given as needed to maintain anesthesia. Animals were sacrificed by anesthetic overdose with intraperitoneal injection of 250 mg/kg body weight sodium pentobarbital before removing the pulmonary artery. Efficiency of anesthesia was monitored by lack of withdrawal reflex upon hind toe pinching, regular respiratory rate 30% below normal and no reaction to skin pinch over the area to be incised. Rat brain endothelial cells were isolated from cortex of Sprague–Dawley pups (7–10 days) and cultured in F-10 Nutrient Mixture containing 16% fetal bovine serum, endothelial cell growth supplement, and other components (heparin, glutamine, gentamicin) as described elsewhere [21]. After culturing for 3–4 weeks, cells were dissociated from plates with trypsin/EDTA, replated at a density of 0.3 million cells/well (approximately 169,500 cells/cm^2^) onto poly-L-lysine-coated 24-well culture plates (Costar), and grown at 37° C, in 5% CO_2_ incubators. Replated brain endothelial cells were grown for 3 days before use. Approximately 98–99% of the cells in these cultures were positive when stained with anti-von Willebrand Factor (vWF related antigen, Santa Cruz Biotechnology) and were negative when stained for the astrocyte marker glial fibrillary acidic protein (GFAP). The total number of cells in the wells was determined by counterstaining nuclei with DAPI, as described elsewhere [7], to calculate percentage of antibody-positive cells. For both vWF and GFAP staining, control wells using secondary antibodies, but lacking primary antibodies, were negative.

### Drug treatment

Prior to stimulation with LPS, BMVECs were incubated with treatment medium consisting of neuronal culture media (NCM) and bovine serum albumin (BSA), then treated with LPS, NG-nitro-L-arginine methyl ester (L-NAME), aminoguanidine (AG), sodium nitroprusside (SNP), WHI-P154 (WHI, an inhibitor of JAK3), fludarabine (Flu, a specific inhibitor of STAT1), or other compounds as previously described [15]. At the end of the incubation/treatment time, the cell culture medium was removed or reserved. Media were assayed within 1 h of collection time for nitrite assays, and the remaining medium was stored at −80 °C until determination of sPLA_2_ enzyme activity and expression levels of sPLA_2_-IIA protein by Western blot.

### Transfection with small interference RNA (siRNA) for STAT1

After reaching 50% confluence, BMVECs (2 × 10^5^ cells/well) were transfected with STAT1 shRNA [STAT1 p84/p91 shRNA (r) lentiviral particles: sc-61879-V] according to the manufacturer’s protocol from Santa Cruz Biotechnology Inc. Transfection complexes were prepared using siRNA reagents, transfection medium, and STAT1 siRNA, and delivered to cell monolayers with a 100 nmol/l final concentration of STAT1 siRNA duplexes. A scrambled control shRNA for STAT1 (sc-108080) was used as a negative control. The effectiveness of STAT1 shRNA was assessed with RT-PCR and Western blot.

### Measurement of Nitrite

Synthesis of NO was determined by assaying 250 μl of the culture media from BMVECs for nitrite (a stable breakdown product of NO) after treatment by LPS and other drugs for 24 h by reaction with Griess reagent (Cayman Chemical) as described previously [2].

### sPLA_2_-IIA production assay

The sPLA_2_-IIA protein released into the MVECs medium was determined using specific enzyme-linked immunosorbent assay (ELISA) kits according to the manufacturer’s instructions (Cayman Chemical) with a minor modification. Briefly, additional standard probes with a concentration of 4 pg/ml or 8 pg/ml, and a long exposure to Ellman’s reagent of at least 4 h were applied to increase the sensitivity of the assay. Total cell protein was determined using a bicinchoninic acid assay kit with bovine serum albumin as the internal standard (Sigma-Aldrich). We found that 12.6 pg of sPLA_2_-IIA was released by 10^6^ brain MVECs (12.6 pg/mg of cell protein) in normal conditions.

### sPLA_2_ enzyme activity assay

The sPLA_2_ enzyme activity was measured using a fluorometric assay, as described elsewhere (15, 22), and shown to be selective for sPLA_2_. The fluorescent substrate 1-hexadecanoyl-2-(1-pyrenedecanoyl)-sn-glycero-3-phosphoglycerol ammonium salt (Molecular Probes) was dried under nitrogen and suspended in ethanol at a concentration of 0.2 mM. Vesicles were prepared by adding the phospholipid substrate to an aqueous buffer solution containing 50 mM Tris–HCl, 500 mM NaCl, and 1 mM EDTA (pH 7.5). Substrate (2 μM final concentration), bovine serum albumin solution, CaCl_2_, and 50 μl of the sample (cell culture medium) were added to the reaction solution as described elsewhere [15], and mixed well. Fluorescence of the reaction medium (blank) was recorded with a Photon Technology International spectrofluorometer (Lawrenceville, NJ) and compared to sample values and activity in pmoles/ml/min derived from the formula described using 5 μg of bee venom phospholipase A_2_ (Cayman Chemical) to establish maximal fluorescence values (F_max_) [15].

### Immunostaining

BMVECs were fixed with acid/ethanol (for the von Willebrand factor) with Diff-Quik [for GFAP(2) or vimentin] or formaldehyde (for sPLA_2_-IIA) [21,22] for 20 min and washed with PBS. Cells were permeabilized with 0.2% or 0.1% Triton X-100 in PBS for 2 min at room temperature and washed three times with 0.1% Triton X-100 in PBS (solution A). The cells were blocked with 5% appropriate serum diluted in 0.1% Triton/PBS overnight at 4° C. Then, the cells were incubated with the primary antibodies diluted in PBS containing 0.1% Tween-20 and 1% bovine serum albumin (solution B) overnight at 4° C. Primary antibodies were anti-sPLA_2_ monoclonal antibody (Cayman), anti-GFAP monoclonal antibody (Sigma), and anti-vimentin (Santa Cruz) used at a dilution of 1:400. After washing four times with solution A, the cells were incubated with secondary antibodies diluted 1:200 in solution B. Secondary antibodies conjugated to FITC or Alexa-488 were added to cells for 0.5-1 h, after which cells were then washed four times with solution A and three times with PBS.

Cells were imaged with a Nikon Diaphot 200 inverted fluorescence microscope and a Hamamatsu color chilled 3CCD camera (Bridgewater, NJ, USA) using Metamorph software (Universal Imaging, PA, USA) on a Windows-based computer with a FlashBus frame grabber (Integral Technologies, Indianapolis, IN, USA).

#### Western blotting

Western blotting analysis was carried out using an XCell SureLockTM Mini-Cell system (Invitrogen Corporation, Carlsbad, CA, USA) as previously described [15]. Blotted membranes were incubated with primary polyclonal antibodies to sPLA_2_-IIA (Cayman), iNOS (Sigma), phospho-STAT1 Tyr701 and STAT1 (Cell signaling), and monoclonal antibody to β-actin and BSA (Sigma), and incubated with secondary antibody for 1 h at room temperature, followed by enhanced chemiluminescence detection (ECL plus, Amersham, Buckinghamshire, England) and exposure to ECL Hyperfilm (Amersham).

### Reverse transcription-polymerase chain reaction (RT-PCR) and real-time PCR

Total RNA was extracted from confluent BMVEC cultures using TRIZOL reagent. The quality and quantity of extracted RNA of BMVECs were determined by NanoDrop 2000 spectrophotometry (Thermo scientific, Wilmington, DE, USA). Reverse transcription of RNA, amplification, detection of DNA, data acquisition, primer design, and quantitative real-time PCR analysis were all performed as described [23]. PCR primers for rat sPLA_2_-IIA, iNOS, and β-actin were as follows: sPLA_2_-IIA: sense, 5’-CAT GGCCTTTGGCTCAATTCAGGT-3’; antisense, 5’-ACAGTCATGAGTCACACAGCACCA-3’; iNOS: sense, 5'-GGAGAGATTTTTCACGACACCC-3', antisense, 5'-CCATGCATAATTTGGACTTGCA-3'; β-actin: sense, 5'- TGAAGATCAAGATCATTGCTCCTCC-3', antisense, 5'-CTAGAAGCATTTGCGGTGGACGATG -3'. The cDNA synthesis reaction was amplified for 38 cycles at 94 °C for 1 min, 58 °C for 1 min, and 72 °C for 2 min as a standard project by PTC-100® Peltier Thermal Cycler (Bio-Rad Laboratories, Inc., USA). For real-time PCR, the thermocycler programs were 95 °C for 2 min followed by 30 cycles of 95 °C for 30 s, 58 °C for 45 s, and 72 °C for 1 min. Melt Curve Analysis was performed at the end of each experiment to verify that a single product per primer pair was amplified. All quantification was normalized to β-actin endogenous control. The amplification and analysis were performed using an iCycler IQ Multicolor Real-Time PCR Detection System (Bio-Rad). The real-time PCR data were quantified using the relative quantification (2^-ΔΔCT^) method.

### Electrophoretic mobility shift assays (EMSA)

Cells were washed in cold PBS, lysed in buffer (15 mM KCl, 10 mM HEPES, pH 7.6, 2 mM MgCl_2_, 0.1 mM EDTA, 1 mM DTT, 0.1% Nonidet P-40, 0.5 mM PMSF, 2.5 μg/ml leupeptin, 5 μg/ml antipain, and 5 μg/ml aprotinin) for 10 min on ice, and centrifuged at 14,000 g for 20 s at 4 °C. Proteins in the nuclei were extracted by incubation at 4 °C with vigorous vortex in buffer A (420 mM NaCl, 20 mM HEPES, pH 7.9, 0.2 mM EDTA, 25% glycerol, 1 mM DTT, 0.5 mM PMSF, 2.5 μg/ml leupeptin, 5 μg/ml antipain, and 5 μg/ml aprotinin) followed by centrifugation at 13,000 g for 30 min at 4 °C. The supernatant extract was collected and stored at −80 °C. The probes were double-stranded oligonucleotides containing a STAT1 consensus oligonucleotide (5'-CATGTTATGCATATTCCTGTAAGTG-3'; Santa Cruz Biotechnology, Santa Cruz, CA) and end-labeled with [γ-^32^P]-ATP (Yahui Biological and Medical Engineering, Beijing). DNA binding reactions were performed in a 25 μl reaction mixture containing 6 μl of nuclear extract (1 mg/ml) and 5 μl of 5× binding buffer (20% Ficoll, 50 mM HEPES, pH 7.9, 5 mM EDTA, and 5 mM DTT). The remainder of the reaction mixture contained 50 mM KCl, 0.1% Nonidet P-40, 1 μg of poly (dI-dC), and 200 pg of the probe. Samples were separated through 5.5% polyacrylamide gels and then exposed to x-ray film.

### Measurement of brain microvascular endothelial cell monolayer permeability

The permeability of BMVEC monolayers was measured by diffusion of biotinylated bovine serum albumin (biotin-BSA). Permeability assays to assess brain barrier function of monolayers were performed using a modified protocol described by Li et al. [24,25]. BMVEC monolayers were seeded (10^5^ cells per insert) on 12-well cell-cultured dishes (Costar, Cambridge, MA) lined with polycarbonate filters (pore size 0.4 μm). The filters were treated for 20 min with 0.1% acetic acid, then for 1 h with 0.1% gelatin, and air-dried before seeding cells. Half of the medium in the wells was changed every day. Usually, monolayer cell forms were monitored for 6 to 8 days post-seeding. The F-10 serum was removed for a period of 24 h prior in studies of monolayer permeability. The upper chamber was filled with 0.5 ml of appropriate F-10 media. Sufficient medium was added to each lower chamber to cover the membrane. When confluent, one group of cultures was infected with control and STAT1 siRNA for 2 days. Select cultures were then treated with LPS with 5 μg/ml for 16 h. S3319 (2 μM), LY311727 (10 μM), L-NAME (1 mM), AG (1 mM), SNP (1 μM), WHI (10 μM), and Flu (50 μM) were added to the upper chamber wells simultaneously with 500 μg/ml biotin-BSA. One hundred microliter aliquots of lower chamber media were aspirated at 0.5 h, and biotin-BSA concentrations were determined by enzyme-linked immunosorbent assay.

### Statistical analysis

Statistical comparisons were performed using the paired, two-tailed Student's *t*-test for experiments consisting of two groups and the one-way ANOVA with the multiple comparison method for experiments consisting of more than two groups. Data are presented as the mean ± SE. The results were considered statistically significant when *P* < 0.05.

## Results

### Characterization of rat BMVECs

**Figure 1:**
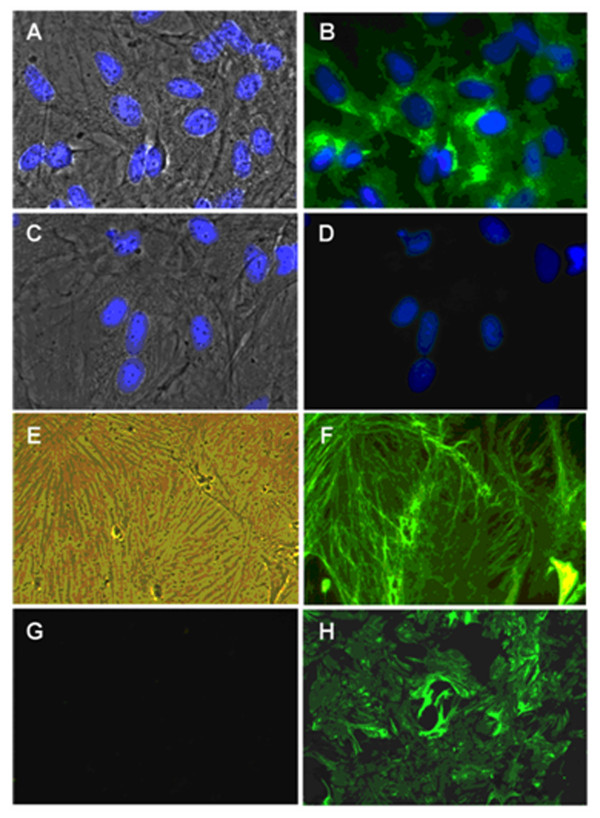
**Characterization of BMVECs by von Willebrand Factor (VWF) and vimentin.** (**A–B**). BMVECs were stained for VWF: phase image + DAPI (blue) shown in panel A; VWF (green) + DAPI (blue) shown in panel B. (**C–D**) Identical BMVEC cultures were grown with the same secondary antibody, only lacking the VWF primary antibody: phase image + DAPI (blue) shown in panel C; no primary antibody + DAPI (blue) shown in panel D. (**E–F**) Phase images and fluorescent images of BMVECs stained for vimentin. (**G**) BMVECs lacking staining for the astrocyte marker GFAP. (**H**) Positive staining for GFAP on astrocyte cultures. Original microscope magnification = 600 × for panels A–D, 400 × for E and F, and 200 × for panels G and H.

Rat BMVECs were isolated and cultured as described in Methods. These cells were shown to abundantly express von Willebrand Factor (vWF), a protein specific to endothelial cells (Figure 1B). The cells were stained with an anti-vimentin antibody (Figure 1F). The cells were also probed with anti-GFAP antibodies, which are astrocyte-specific markers, and no expression was detected (Figure 1G). The results showed that over 98% of the cells were vWF-positive. Astrocytes served as GFAP-positive controls (Figure 1H).

### Release of sPLA_2_-IIA from rat BMVECs after LPS stimulation

**Figure 2:**
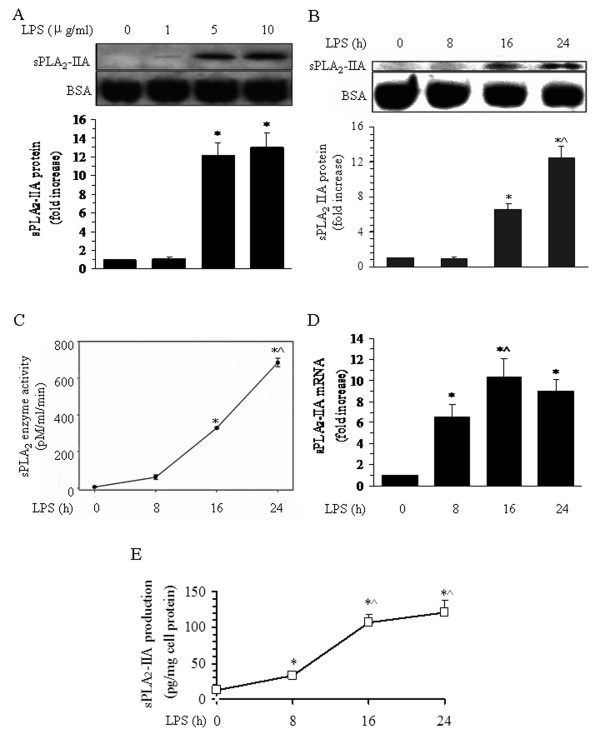
**Dose and time course of sPLA**_**2**_**-IIA release into BMVEC culture medium.** BMVECs were treated with 0, 1, 5, or 10 μg/ml of LPS as described in the Methods, and the cell culture medium was collected to determine the sPLA_2_-IIA protein and mRNA expression by Western blotting and real-time PCR. The same blotted membrane was stripped and re-probed for BSA, and the relative sPLA_2_-IIA band intensity was calculated. (**A**) Representative bands showing the dose response curves of sPLA_2_-IIA protein (upper panel) and BSA (middle panel) from Western blotting. Quantitative data of sPLA_2_-IIA release after LPS treatment for 24 h are shown in the lower panel. The data are presented as the means ± SE of the four separate experiments. *P < 0.05 versus the normal group (LPS 0 μg/ml) and LPS treatment with 1 μg/ml. (**B**) Representative bands showing the Western blotting results for sPLA_2_-IIA protein (upper panel) and BSA (middle panel). Quantitative data of sPLA_2_-IIA release after LPS treatment are shown in the lower panel. The data are presented as the means ± SE of the four separate experiments. *P < 0.05 versus the normal group (time 0 h) and LPS treatment for the 8-h group, ^^^*P* < 0.05 versus LPS treatment for the 16-h group. (**C**) A line chart shows representative results of sPLA_2_ enzyme activity in the culture medium after 5 μg/ml LPS treatment. The data shown are the averages of four separate experiments from four individual platings (means ± SE). *P < 0.05 versus the normal group (time 0 h) and LPS treatment for the 8-h group, ^^^*P* < 0.05 versus LPS treatment for the 16-h group. (**D**) Quantitative data of sPLA_2_-IIA mRNA expression after LPS treatment with 5 μg/ml for different times. The data are presented as the means ± the SE of the four separate experiments. *P < 0.05 versus the normal group (time 0 h), ^^^*P* < 0.05 versus LPS treatment for the 8-h group. (**E**) Quantitative data of sPLA_2_-IIA release into the MVECs medium after 5 μg/ml LPS treatment by ELISA. The data are presented as the means ± the SE of the four separate experiments. *P < 0.05 versus the normal group (time 0 h), ^*P* < 0.05 versus the LPS 8-h group.

When treated with 5 or 10 μg/ml of the inflammatory stimulus LPS, BMVECs were found to release increased amounts of sPLA_2_-IIA protein into the cell culture medium (Figure 2A). These increases were first detectable at 8 h by ELISA and at 16 h by Western blotting. Quantitative results showed that sPLA_2_-IIA protein levels increased 6.2-fold at 16 h relative to the control or the levels at 8 h. Between 16 and 24 h, sPLA_2_-IIA protein levels increased by 2-fold (Figure 2B). Identification of this protein as a sPLA_2_ was supported by enzyme analysis of the BMVEC culture medium. The sPLA_2_ enzyme activity increased to 5.2-fold between 8 (66.3 pM/ml/min) and 16 h (345.5 pM/ml/min) after LPS treatment and doubled again by 24 h (696.4 pM/ml/min, Figure 2C). The levels of sPLA_2_-IIA in the cell medium reached to 2.6-, 8.5-, and 9.6-fold at 8, 16, and 24 h, respectively, relative to the normal group (time 0 h) (Figure 2E). These results show that LPS induces the release of sPLA_2_-IIA protein from rat BMVECs in a time- and dose-dependent manner.

### Effects of NOS inhibitor pretreatment on sPLA_2_-IIA expression in BMVECs

**Figure 3:**
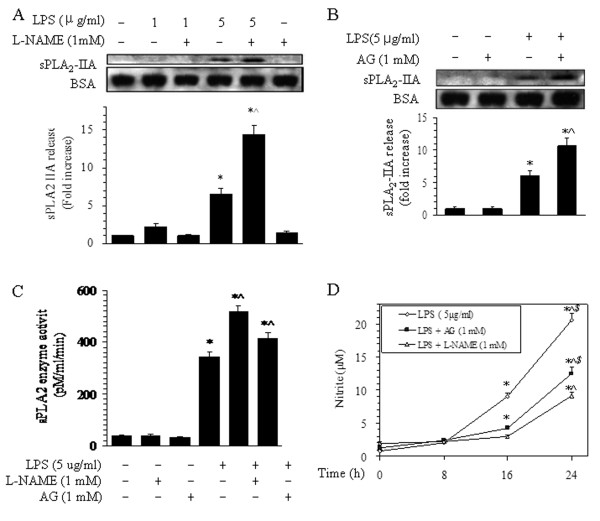
**Effects of L-NAME and aminoguanidine (AG) on the release of sPLA**_**2**_**-IIA from BMVECs.** (**A**) Representative bands showing the sPLA_2_-IIA (upper panel) and BSA (middle panel) from the medium from BMVECs pretreated with L-NAME. Brain endothelial cells were treated for 16 h with 1 or 5 μg/ml of LPS in the presence or absence of L-NAME as indicated, and Western blots were performed on the culture medium using an antibody against sPLA_2_-IIA. Quantitative data on the release of sPLA_2_-IIA from BMVECs are shown in the lower panel. The data are presented as the means ± SE of the three separate experiments. *P < 0.05 versus the normal group, ^^^*P* < 0.05 versus the group without L-NAME. (**B**) Representative bands showing the sPLA_2_-IIA (upper panel) and BSA (middle panel) from the medium from BMVECs pretreated with AG. The cells were treated for 16 h with 5 μg/ml of LPS in the presence or absence of 1 mM AG, as indicated, and Western blots were performed on the culture medium using an antibody against sPLA_2_-IIA. Quantitative data on the release of sPLA_2_-IIA from BMVECs are shown in the lower panel. The data are presented as the means ± SE of the three separate experiments. *P < 0.05 versus the normal group and AG-alone group, ^#^*P* < 0.05 versus the group without AG treatment. (**C**) A histogram showing the representative results of sPLA_2_ enzyme activity in the culture medium after 5 μg/ml LPS treatment for 16 h and pretreatment with L-NAME and AG. The data shown here are the averages of four separate experiments from four individual platings (means ± SE). *P < 0.05 versus the normal group, ^^^*P* < 0.05 versus LPS treatment alone. (**D**) Time course of nitrite production in BMVECs after LPS treatment with or without L-NAME and AG. Upper line: Nitrite concentration from the medium from BMVECs treated with LPS alone for different times (0, 8, 16, 24 h). The culture medium from BMVECs collected at the indicated times. Middle line: BMVECs were treated with LPS in the presence of L-NAME for 15 min prior to LPS stimulus, the culture medium was collected at the indicated times, and nitrite concentrations were calculated. Bottom line: BMVECs were treated with LPS in the presence of AG 15 min prior to LPS stimulus, the culture medium was collected at the indicated times, and nitrite concentrations were calculated. The data shown are the averages of five separate experiments from six separate platings; *P < 0.05 versus their respective normal groups (time 0 h) and 8-h treatment groups, ^^^*P* < 0.05 versus their respective 16-h LPS treatment groups, ^$^*P* < 0.05 versus treatment with LPS alone.

The effects of pretreatment with the NOS inhibitors L-NAME and AG on LPS-induced sPLA_2_-IIA expression in BMVECs were analyzed with Western blotting and enzyme activity assays. Pretreatment of cells with 1 mM of the NOS inhibitor L-NAME for 15 min before 16 h of LPS stimulation caused a 2.2-fold increase in the LPS-stimulated release of sPLA_2_-IIA relative to treatment with LPS alone (5 μg/ml) (Figure 3A). After pretreatment with 1 mM of the inducible NOS inhibitor AG for 15 min, sPLA_2_-IIA protein levels in the culture medium increased 1.7-fold relative to LPS alone (5 μg/ml) (Figure 3B). The sPLA_2_ enzyme activity level in the L-NAME pretreatment group was augmented 1.5-fold (518.2 pM/ml/min), and the level in the AG pretreatment group increased to 1.2-fold (414.6 pM/ml/min), compared with LPS treatment alone (5 μg/ml) (345.5 pM/ml/min, Figure 3C). These results show that L-NAME and AG pretreatment potentiates LPS-stimulated sPLA_2_-IIA release from BMVECs.

Treatment of BMVECs with LPS also caused increased generation of nitrite, a stable metabolite of nitric oxide, which was detected in the culture medium (Figure 3D). Nitrite levels improved to 12.1-fold (9.08 μM) at 16 h, compared with the normal group (0.75 μM). Levels at 24 h were 2.3-fold (20.55 μM) higher than those at 16 h. Pretreatment with 1 mM of L-NAME or AG before LPS stimulation caused nitrite levels to decrease by 38.7% (5.56 μM) and 67.5% (2.95 μM), respectively, compared with LPS treatment alone at 16 h. Nitrite levels after treatment with L-NAME or AG were attenuated by 31.4% (14.10 μM) and 55.4% (9.17 μM), respectively, compared with LPS treatment alone at 24 h (Figure 3D). These results show that pretreatment with NOS inhibitors delay and attenuate the generation of nitrite from BMVECs stimulated with LPS. Together, these results show that NOS inhibitor pretreatment can augment LPS-stimulated sPLA_2_-IIA release via the inhibition of nitrite production by BMVECs.

### Effects of NO donor-induced nitrite accumulation on the release of sPLA_2_-IIA

**Figure 4:**
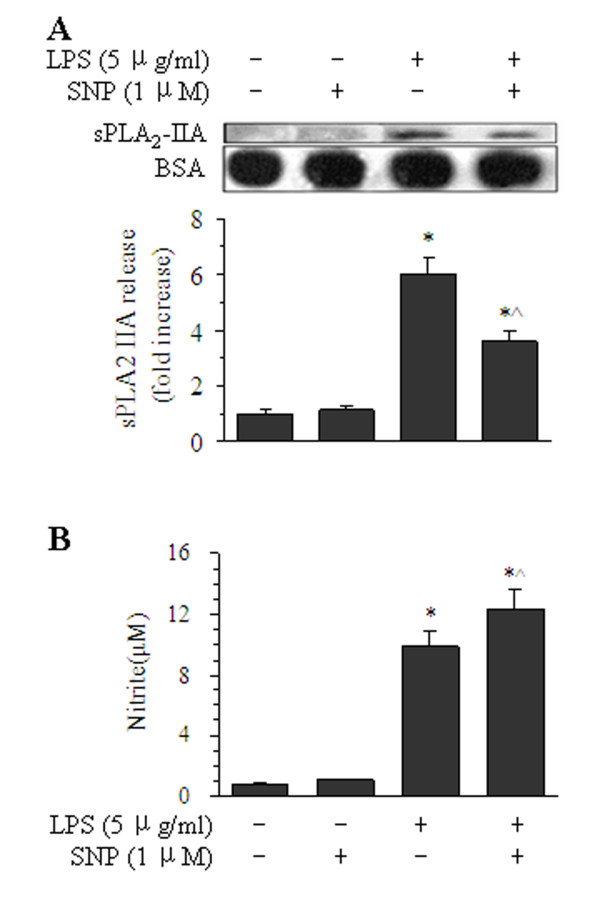
**Effects of sodium nitroprusside (SNP) on the release of sPLA**_**2**_**-IIA from BMVECs.** BMVECS were treated with 5 μg/ml of LPS for 16 h, and the culture medium was Western blotted with antibodies against sPLA_2_-IIA. (**A**) Cells were pretreated with or without 1 μM of SNP for 15 min before LPS stimulus. The blots shown are representative of one of three separate experiments, the same membrane was re-probed for BSA, and the relative sPLA_2_ band intensity was calculated as shown in the bar graph below the blot. (**B**) Corresponding nitrite levels for each condition by LPS treatment with SNP. The data shown are the averages of four separate experiments (means ± SE), *P < 0.05 versus their respective normal groups, ^^^*P* < 0.05 versus their respective LPS alone groups.

Pretreatment of BMVECs with the NO donor SNP at 1 μM diminished the release of LPS-induced sPLA_2_-IIA by 39.9%, compared with LPS treatment alone for 16 h (Figure 4A). In contrast, nitrite production increased to 1.3-fold (12.34 μM), compared with LPS treatment alone for 16 h (9.86 μM, Figure 4B). These results show that SNP-induced nitrite accumulation can inhibit the release of sPLA_2_-IIA from BMVECs.

### Regulation of nitrite and iNOS expression by STAT1 siRNA and inhibitors of JAK3 and STAT1 after LPS stimulation

**Figure 5:**
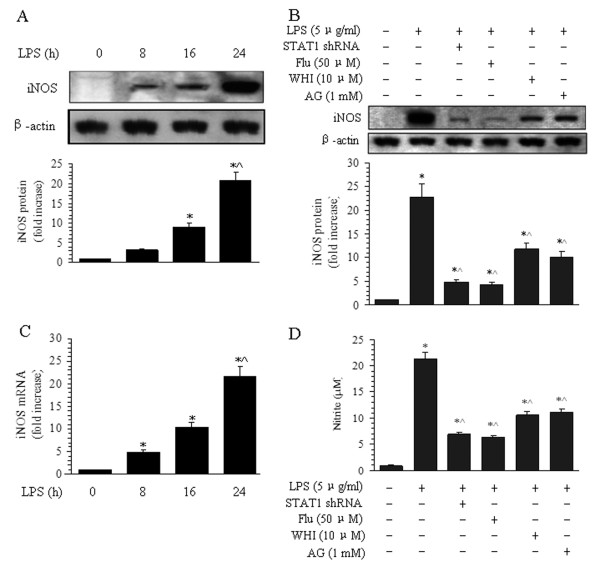
**Effects of the inhibitors of iNOS, JAK3, and STAT1 on the expression of iNOS in BMVECs after LPS treatment.** (**A**) Time course of iNOS protein expression in BMVEC cells. BMVECs were treated with 5 μg/ml of LPS as described in Methods, and cells were lysed to determine the iNOS protein levels by Western blotting. The same blotted membrane was stripped and re-probed for β-actin, and the relative intensity of the iNOS band was calculated. Representative bands (upper panel) show the Western blotting results for iNOS protein (upper bands) and beta-actin (lower bands). Quantitative data of iNOS expression after LPS treatment are shown in the lower panel. The data are presented as the means ± SE of four separate experiments. *P < 0.05 versus the normal group (time 0 h), ^^^*P* < 0.05 versus LPS treatment for 16-h group. (**B**) Effects of inhibitors of JAK3, STAT1, and iNOS on the expression of iNOS. Representative bands (upper panel) show the Western blotting results for iNOS protein (upper bands) and beta-actin (lower bands) by pretreatment with STAT1 siRNA, Flu, WHI, and AG. Quantitative data of iNOS expression after inhibitor treatment are shown in the lower panel. The data are presented as the means ± SE of four separate experiments. *P < 0.05 versus the normal group (time 0 h), ^^^*P* < 0.05 versus LPS treatment alone. (**C**) Time course of iNOS mRNA expression in BMVEC cells by real-time PCR methods. The data shown are the averages of four separate experiments from four individual platings (the means ± SE). *P < 0.05 versus the normal group (time 0 h), ^^^*P* < 0.05 versus the 16-h LPS treatment group. (**D**) Effects of inhibitors of JAK3 and STAT1 on nitrite production by BMVECs after LPS and inhibitor treatment. Quantitative data of nitrite production from BMVECs after inhibitor treatment are shown in the histogram. The data are presented as the means ± SE of six separate experiments. *P < 0.05 versus the normal group, ^#^*P* < 0.05 versus LPS treatment alone.

Effects of the inhibitors of JAK3 (WHI), STAT1 (Flu), and NOS (AG) on iNOS expression were tested by Western blot (Figure 5). Treatment of BMVECs with LPS also increased the generation of iNOS protein (Figure 5A). At 8, 16, and 24 h, the levels of iNOS were 3.2-, 8.9-, and 20.7-fold greater than those of the normal control group. At 24 h, iNOS levels were 2.3-fold greater than those at 16 h. Consistent with these results, the mRNA expression of iNOS was increased in a time-dependent manner as determined by real-time PCR (Figure 5C).

Pretreatment of cells with 1 mM of AG for 15 min before LPS stimulation lasting 24 h caused iNOS protein levels to decrease to 48.9% of those observed in cells treated with LPS alone (Figure 5B). Similarly, NO levels went down under AG pretreatment conditions. This shows that nitrite production is regulated, at least in part, by iNOS expression (Figure 5D).

Pretreatment with STAT1 siRNA, Flu, or WHI before LPS stimulation lasting 24 h caused iNOS protein levels to diminish to 23.5%, 21.0%, and 57.1%, respectively, of the protein levels observed after LPS treatment alone (Figure 5B). Pretreatment with STAT1 siRNA, Flu, or WHI caused the release of nitrite from BMVECs to decline to 33.6% (6.9 μM), 28.7% (5.9 μM), and 51.6% (10.6 μM), respectively, of the levels observed after LPS treatment alone (20.55 μM, Figure 5D). These results show that inhibition of JAK3 and STAT1 can suppress iNOS expression, which, in turn, reduces the production of nitrite from BMVECs after LPS stimulation.

### Regulation of nuclear STAT1 activation and STAT1 phosphorylation by STAT1 siRNA and inhibitors of JAK3 and STAT1

**Figure 6:**
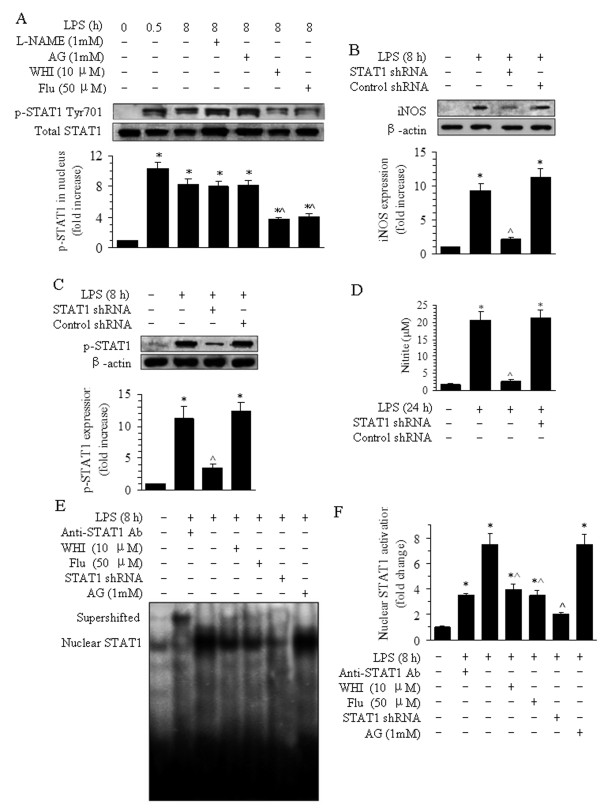
**Effects of L-NAME, AG, WHI, Flu, and STAT1 siRNA on the phosphorylation of STAT1 and nuclear STAT1 expression in BMVECs after LPS treatment.** (**A**) Effects of L-NAME, AG, WHI, and Flu on the phosphorylation of STAT1 Tyr701 in BMVECs were detected using Western blotting. The data are presented as the means ± SE of four separate experiments. *P < 0.05 versus the normal group (time 0 h), ^^^*P* < 0.05 versus LPS alone group (8 h). (**B**) The effects of STAT1 siRNA on the phosphorylation of STAT1 Tyr701 in BMVECs were detected. The data are presented as the means ± SE of the three separate experiments. (**C**) Effects of STAT1 siRNA on the release of nitrite from BMVECs after LPS treatment for 24 h. The data are presented as the means ± SE of five separate experiments. *P < 0.05 versus the normal group (time 0 h). ^*P* < 0.05 versus LPS treatment alone group. (**D**) Nitrite production modulated by STAT1. (**E**) Representative bands showing the EMSA results for the activation of nuclear STAT1 in BMVECs after pretreatment with WHI, Flu, siRNA STAT1, AG, or anti-STAT1 antibodies. (**F**) Quantitative data of the activation of nuclear STAT1 after inhibitor treatment are shown in the histogram. The data are presented as the means ± SE of four separate experiments. *P < 0.05 versus the normal group (time 0 h), ^^^*P* < 0.05 versus LPS treatment alone groups.

Treatment of BMVECs with LPS for 0.5 and 8 h caused phosphorylation of STAT1, which was detected in the cells by Western blotting. LPS treatment also caused 10.3- and 8.3-fold increases at 0.5 and 8 h, respectively, compared with the normal group at 0 h (Figure 6). Pretreatment with WHI or Flu decreased STAT1 phosphorylation in the BMVECs to 44.4% and 49.5%, respectively, of the levels observed after LPS treatment alone (Figure 6A). When the cells were transfected with STAT1 siRNA, the phosphorylation of STAT1 decreased, and subsequently, the iNOS protein levels also dropped. Consistent with the STAT1 phosphorylation, the nitrite production from BMVECs decreased to approximately 22% (4.68 μM) of that observed in the normal group (20.55 μM) and the scrambled STAT transfection group (21.97 μM) (Figure 6B, C and D). In contrast, pretreatment with 1 mM of L-NAME or AG before LPS stimulus did not have any clear effects on the phosphorylation of STAT1 in BMVECs (Figure 6A). These results show that interference of JAK3 and STAT1 can suppress the phosphorylation of STAT1 in BMVECs and inhibit the release of nitrite from the cells.

Treatment of BMVECs with LPS also caused nuclear STAT1 activation in BMVECs, which was detected in the cells by EMSA. Levels of nuclear STAT1 activation were increased 7.5-fold at 8 h, compared with the 0 h. Pretreatment with WHI or Flu also decreased STAT1 activity in the nucleus of BMVECs to 53.1% or 46.7%, respectively, compared with LPS treatment alone. Nuclear STAT1 activity dropped to 26.9% in the cells pre-transfected with STAT1 siRNA. In contrast, pretreatment of cells with 1 mM AG before LPS stimulation did not produce any clear effect on the nuclear activity of STAT1 in BMVECs (Figure 6E and F). These results show that inhibition of JAK3 and STAT1 can regulate the LPS-induced nuclear activity of STAT1 in BMVECs.

### Effects of STAT1 siRNA and inhibitors of JAK3 and STAT1 on the release of sPLA_2_-IIA after LPS stimulation

**Figure 7:**
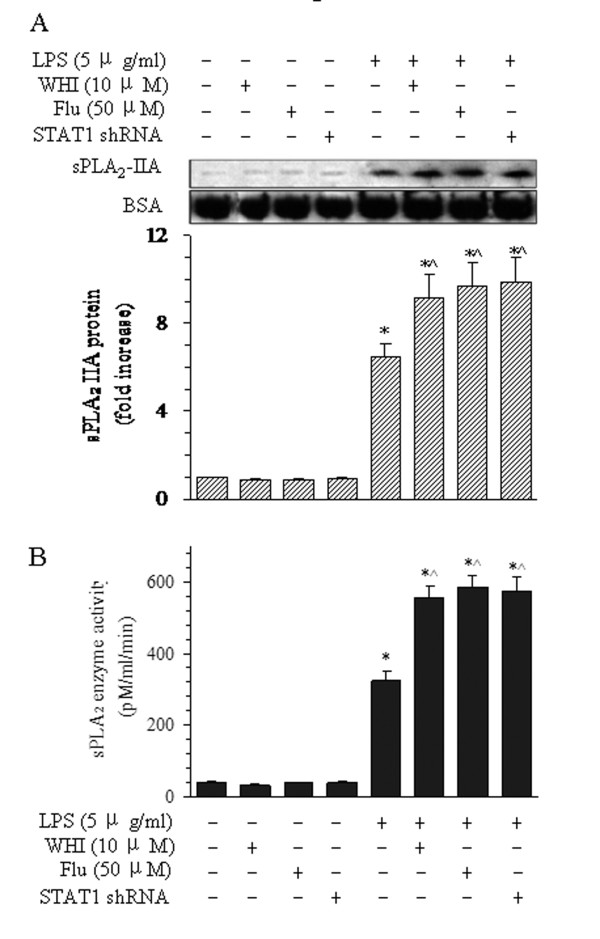
**Effects of WHI, Flu, and siRNA STAT1 on the release of sPLA**_**2**_**-IIA from BMVECs and enzymatic activity of sPLA**_**2**_**.** (**A**) Effects of WHI, Flu, and siRNA STAT1 on the release of sPLA_2_-IIA from the cells after LPS treatment for 16 h. Representative bands (upper panel) are shown of the Western blotting results for sPLA_2_-IIA and BSA protein in the medium after LPS treatment with WHI and Flu or STAT1 siRNA. (**B**) Quantitative data of sPLA_2_ enzyme activity after LPS and treatment with WHI, Flu, and siRNA STAT1 are shown in the histogram. The data are presented as the means ± SE of five separate experiments. *P < 0.05 versus the normal group, ^^^*P* < 0.05 versus LPS treatment alone group.

With pretreatment of BMVECs with WHI, flu, or STAT1 siRNA, the protein levels of sPLA_2_-IIA were augmented to 1.6-, 1.7-, and 1.8-fold, respectively, compared with those after LPS treatment alone for 16 h (Figure 7A). Consistent with these findings, pretreatment with WHI, Flu, or STAT1 siRNA also increased sPLA_2_ enzyme activity to 1.5- (517.5 pM/ml/min), 1.6-fold (545.9 pM/ml/min), and 1.6-fold (539.1 pM/ml/min), respectively, compared with LPS treatment alone (345.5 pM/ml/min) (Figure 6B). These results show that inhibition of JAK3 and STAT1 can enhance sPLA_2_-IIA protein expression in BMVECs.

### Effect of inhibitors of sPLA_2_-IIA, NO and STAT1 on the monolayer permeability of BMVECs

**Figure 8:**
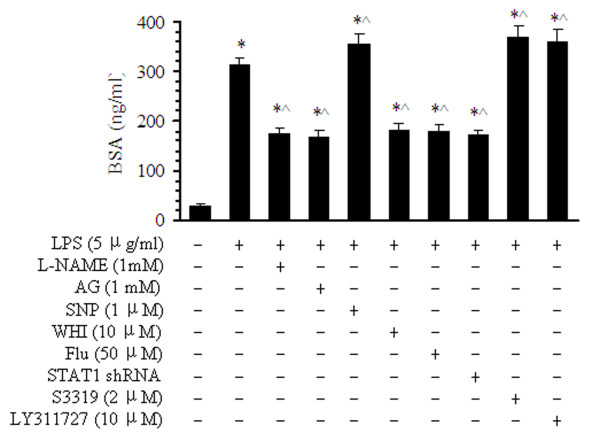
**Effects of sPLA**_**2**_**-IIA inhibitor, NOS inhibitors, and STAT1 siRNA on the permeability of BMVECs after LPS treatment.** Cells were pretreated with the sPLA_2_-IIA inhibitors S3319 (2 μM) and Ly311727 (10 μM); the NOS inhibitors L-NAME (1 mM) and AG (1 mM); the NOS agonist, SNP (1 μM); JAK3 (10 μM); the STAT1 inhibitors, WHI (10 μM) and Flu (10 μM); and STAT1 siRNA; and then the BMVECs were stimulated by 5 μg/ml LPS for 16 h. BMVEC permeability was analyzed by the effusion capacity of biotin-BSA from the cell monolayers. Quantitative data of the cell permeability after LPS and pretreatment with L-NAME, AG, SNP, WHI, Flu, or siRNA STAT1. The data are presented as the means ± SE of five separate experiments. *P < 0.05 versus the normal group, ^^^*P* < 0.05 versus LPS treatment alone group.

To determine the effect of sPLA_2_-IIA, NO, and STAT1 inhibitors on the monolayer permeability of BMVECs, cells were infected with scrambled or STAT1 siRNA lentivirus, or pretreated with L-NAME, AG, SNP, WHI, or Flu. Next, cells were treated with LPS at 5 μg/ml for 16 h. The biotin-BSA concentrations increased to 10.6-, 5.9-, 5.7-, 6.2-, 6.1-, and 5.8-fold in the LPS treatment alone and pretreatment with L-NAME, AG, WHI, Flu, and siRNA STAT1 groups, respectively, compared with that of the control group (29.4 ng/ml, Figure 8). Pretreatment with L-NAME, AG, WHI, Flu, or STAT1 siRNA decreased these levels to 56%, 54%, 58%, and 57%, respectively, compared with LPS treatment alone (312.2 ng/ml). However, with pretreatment by SNP, S3319, or LY311727, the levels of BSA augmented by 14% (355.6 ng/ml), 18% (368.6 ng/ml), and 15% (359.7 ng/ml), respectively, relative to LPS alone, and by 12-fold relative to the normal control group. Importantly, and consistent with the actions of NO and JAK3/STAT1 signals in the modulation of sPLA_2_ expression, infection of the cells with STAT1 siRNA attenuated LPS-induced permeability mainly through the activation of sPLA_2_. This finding shows that autocrine sPLA_2_-IIA release induced by LPS in low concentrations could protect the cells from LPS-induced injury. These data indicate that sPLA_2_ and NO regulate the monolayer permeability of BMVECs, at least partly, through the JAK3-STAT1 signal pathway.

## Discussion

Our results reveal that sPLA_2_-IIA and nitrite production likely have important regulatory roles in the permeability of BMVECs and the processes of injured brain vessels via the JAK3/STAT1 signal pathway. The following experimental evidence supports this hypothesis: (1) sPLA_2_-IIA protein levels were increased in the media of rat BMVECs after treatment with LPS; (2) secretion of sPLA_2_-IIA from BMVECs was enhanced with the nitrite-diminishing pretreatment with the NOS inhibitors L-NAME or AG before LPS stimulation and inhibited by nitrite pretreatment with the NO donor SNP before LPS stimulation; (3) treatment with LPS also increased the generation of iNOS protein and nitrite, and NOS expression controlled nitrite levels; (4) iNOS expression and nitrite production were regulated by NOS, JAK3, and STAT1 inhibitors in BMVECs after LPS treatment; (5) the release of sPLA_2_ was regulated by JAK3 and STAT1 signaling in BMVECs after LPS stimulus; (6) the permeability of BMVECs was protected by pretreatment with inhibitors of NOS, JAK3, and STAT1 and with STAT1 siRNA. These results demonstrate that in BMVECs after LPS stimulation, the release of sPLA2-IIA is controlled by the nitrite levels, which are regulated, in part, by the JAK3/STAT1 signal pathway.

sPLA_2_-IIA is an active regulator of the BBB and neurovascular units including neurons and glial cells. It has been reported that sPLA_2_-IIA causes apoptosis in neurons in a concentration- and time-dependent manner [26]. The fact that sPLA_2_-IIA can induce neuronal cell death might be associated with NMDA receptor activation and arachidonic acid (AA) metabolites. sPLA_2_ contributes to neurodegeneration in the ischemic brain [27,28]. sPLA_2_-IIA-induced apoptosis has been found to take place in cooperation with the influx of Ca2^+^[29]. The release of sPLA_2_ from brain astrocytes has been found to increase after the cells respond to inflammatory stimuli, such as LPS and cerebral ischemia-reperfusion [30]. Cytokines have been found to induce sPLA_2_-IIA release from astrocytes via oxidative pathways [31]. Here, we show for the first time that rat BMVECs release sPLA_2_-IIA in a time- and dose-dependent manner after LPS stimulation. These results suggest that LPS-induced sPLA_2_-IIA might have an important action in the regulation of the function of the BBB and neurovascular injury.

Our previous study showed that the release of inflammatory sPLA_2_ from the glial cells is regulated by basal nitric oxide levels [15]. Other studies have reported that sPLA_2_ transfection of macrophages increases nitrite production. sPLA_2_ may induce the nitrites and iNOS in the presence of LPS, which is a potent activator of some cells [32,33]. Distinct pathways for the induction of iNOS and sPLA_2_ by cytokines in an immortalized astrocyte cell line (DITNC) have also been demonstrated [34]. The inhibitory effect of ethanol on NO production in astrocytes corresponds with decreases in iNOS protein and NOS enzyme activity, but not with sPLA_2_ mRNA in DITNC cells [35]. Nitric oxide produced by nitric oxide synthase in the endothelium is a key regulator of vascular homeostasis. NO is important for the maintenance of cerebral blood flow after trauma [36]. The relationship between the nitrite/NOS and sPLA_2_ expression in neurovascular cells is still unclear. Here, we show for the first time that the release of sPLA_2_-IIA from BMVECs is regulated by NO as demonstrated by the fact that pretreatment with the NOS inhibitor L-NAME potentiates the LPS-induced release of sPLA_2_-IIA. Post-treatment with L-NAME inhibits the release of sPLA_2_, while pretreatment with low concentrations of the NO donor sodium nitroprusside (SNP) increased sPLA_2_-IIA release, indicating that NO potentially has dual roles in modulating the release of sPLA_2_ (data not shown) from BMVECs. These findings suggest that sPLA_2_/NO is an important mediator of the progress of brain microvascular injury and the BBB.

It has been reported that eNOS is upregulated at the transcriptional level via the action of protein phosphatase 2A, which is activated by a signaling pathway that includes JAK2 and ERK1/2 [37]. p38 MAPKs are required for the synergistic induction of iNOS by LPS and IFN-gamma in murine aortic endothelial cells (MAECs). The synergistic induction of these components is associated with phosphorylation of STAT1 serine 727 in MAECs [38]. The endothelial production of NO was reported to be dependent on adequate cellular levels of tetrahydrobiopterin (BH4), an important cofactor for NOS. Cytokines stimulate the induction of GTP cyclohydrolase I, suggesting the role of STAT3 in modulating STAT1-supported gene transcription [39]. LPS and IFN gamma cause an increase in monolayer permeability and induce the production of iNOS and nitric oxide in a JAK2-dependent manner in MVECs from mice skeletal muscle [40]. RNA silencing of STAT3 blocks the inhibitory effect of IL-6 on endothelial NOS expression in human aortic endothelial cells [41]. The addition of endothelial NOS inhibitors prior to the application of growth hormone (GH) significantly increases the levels of phospho-STAT5b and phospho-JAK2 over the levels observed after GH alone in hepatocytes [42]. In addition, LPS plus IFN gamma-stimulated skeletal muscle MVECs produces ROS that activate the JNK-AP1 and JAK2-IRF1 signaling pathways required for iNOS induction [19]. To our knowledge, this present study is the first to show that NO production and iNOS expression are regulated by JAK3 and STAT1 signal pathways in rat BMVECs after LPS stimulation. Other data have suggested that EPO treatment in intracerebral hemorrhage induces better functional recovery while reducing perihematomal inflammation and apoptosis via activations of eNOS, STAT3, and ERK [43]. One group reported that sPLA_2_ contributes to neurodegeneration in the ischemic brain, which suggests the therapeutic potential of sPLA_2_-IIA inhibitors for stroke [22]. Some reports have shown that L-NAME treatment or inhibition of iNOS can reduce BBB permeability in BMVECs and microvessels in vivo [44,45]. These findings are consistent with our present results. Additionally, the inhibition of JAK3/STAT1 may protect BMVEC permeability.

We also demonstrated, for the first time, that pretreatment of BMVECs with S3319, a specific sPLA_2_-IIA inhibitor, and LY311727, a sPLA_2_ inhibitor, depressed the basal levels of autocrine sPLA_2_ released from LPS-treated BMVECs and can destroy the cell integrity. Autocrine sPLA_2_-IIA release induced by LPS in low concentrations could protect the cells from LPS-induced injury. Trousson demonstrated that the inhibition of sPLA_2_-IIA accelerated apoptosis in oligodendrocytes, and sPLA_2_-IIA partially protected the cells against oxysterol-triggered apoptosis [46]. Others reported on the anti-inflammatory and bactericidal properties of sPLA_2_-IIA and its capability to enhance clearance of oxidative modified lipoproteins during inflammation [47]. This research supports the hypothesis that there are protection effects of sPLA_2_-IIA during cellular inflammation. Certainly, some researchers have thought of sPLA_2_ as an inflammatory factor during certain conditions. The inflammation that occurred during atherosclerosis is characterized by the release of large amounts of sPLA_2_-IIA [48]. Thus, the mechanisms and multiple effects of sPLA_2_-IIA on cells, including BMVECs, need to be further investigated.

Our present research suggests that specific inhibitors of NO, iNOS, JAK3, and STAT1 such as L-NAME, AG, WHI, Flu, or siRNA against STAT1 could serve as potential drugs for the treatment of injured BMVECs. Here, we provide new mechanistic insights into the anti-inflammatory activities of injured BMVECs in the central nervous system and their potential in novel therapeutic strategies for the management of neuroinflammatory diseases.

## Conclusions

Brain microvascular endothelial cells (BMVECs) are the main components of the blood–brain barrier, whose dysfunction plays an important role in the pathological processes of traumatic brain injury and cerebral inflammatory diseases. This study demonstrates that NO and sPLA_2_-IIA can regulate the permeability of BMVECs, and the nitrite production plays an important regulatory role in the secretion of sPLA_2_-IIA from injured BMVECs via the JAK3/STAT1 signal pathway.

## Abbreviations

BBB, blood–brain barrier; NO, nitric oxide; sPLA2-IIA, secreted phospholipase A2-IIA; JAK3 STAT1 LPS, lipopolysaccharide; BMVECs, brain microvascular endothelial cells; iNOS, induced nitric oxide synthase; WHI, WHI-P154; Flu, fludarabine; siRNA, small interfering RNA; L-NAME, NG-nitro-L-arginine methyl ester; AG, aminoguanidine; SNP, sodium nitroprusside; EMSA, electrophoretic mobility shift assays; GFAP, glial fibrillary acidic protein; vWF, von Willebrand factor.

## Competing interests

The authors declare that they have no competing interests.

## Authors’ contributions

GW and MAD designed the study, performed the bulk of the experiments, and analyzed all data. GW, GQ, and CW wrote the manuscript. GW, ZX, JZ, and YW performed the Western blot analysis. PQ, and SC performed the RT-PCR and nitrite analysis. All authors have read and approved the final version of this manuscript.

## References

[B1] Zhou H Andonegui G Wong CH Kubes P Role of endothelial TLR4 for neutrophil recruitment into central nervous system microvessels in systemic inflammation J Immunol 2009 183 5244 5250 10.4049/jimmunol.0901309 19786543

[B2] Balsinde J Winstead MV Dennis EA Phospholipase A (2) regulation of arachidonic acid mobilization FEBS Lett 2002 531 2 6 10.1016/S0014-5793(02)03413-0 12401193

[B3] Six DA Dennis EA The expanding superfamily of phospholipase A(2) enzymes: classification and characterization Biochim Biophys Acta 2000 1488 1 19 10.1016/S1388-1981(00)00105-0 11080672

[B4] Gora S Maouche S Atout R Wanherdrick K Lambeau G Cambien F Ninio E Karabina SA Phospholipolyzed LDL induces an inflammatory response in endothelial cells through endoplasmic reticulum stress signaling FASEB J 2010 24 3284 3297 10.1096/fj.09-146852 20430794

[B5] Connelly L Palacios-Callender M Ameixa C Moncada S Hobbs AJ Biphasic regulation of NF-kappa B activity underlies the pro- and anti-inflammatory actions of nitric oxide J Immunol 2001 166 3873 3881 11238631 10.4049/jimmunol.166.6.3873

[B6] Jones MK Tsugawa K Tarnawski AS Baatar D Dual actions of nitric oxide on angiogenesis: possible roles of PKC, ERK, and AP-1 Biochem Biophys Res Commun 2004 318 520 528 10.1016/j.bbrc.2004.04.055 15120632

[B7] Daniel B DeCoster MA Quantification of sPLA2-induced early and late apoptosis changes in neuronal cell cultures using combined TUNEL and DAPI staining Brain Res Protocols 2004 13 144 150 10.1016/j.brainresprot.2004.04.001 15296851

[B8] Kim DK Fukuda T Thompson BT Cockrill B Hales C Bonventre JV Bronchoalveolar lavage fluid phospholipase A2 activities are increased in human adult respiratory distress syndrome Am J Physio 1995 269 L109 L118 10.1152/ajplung.1995.269.1.L109 7631805

[B9] Sonoki K Iwase M Sasaki N Ohdo S Higuchi S Takata Y Iida M Secretory PLA2 inhibitor indoxam suppresses LDL modification and associated inflammatory responses in TNFalpha-stimulated human endothelial cells Br J Pharmacol 2008 153 1399 1408 18264128 10.1038/bjp.2008.12 PMC2437901

[B10] Haapamaki MM Gronroos JM Nurmi H Soderlund K Peuravuori H Alanen K Nevalainen TJ Elevated group II phospholipase A2 mass concentration in serum and colonic mucosa in Crohn's disease Clin Chem Lab Med 1998 36 751 755 9853800 10.1515/CCLM.1998.133

[B11] Chilton FH Averill FJ Hubbard WC Fonteh AN Triggiani M Liu MC Antigen-induced generation of lyso-phospholipids in human airways J Exp Med 1996 183 2235 2245 10.1084/jem.183.5.2235 8642333 PMC2192563

[B12] Rosenberger TA Villacreses NE Hovda JT Bosetti F Weerasinghe G Wine RN Harry GJ Rapoport SI Rat brain arachidonic acid metabolism is increased by a 6-day intracerebral ventricular infusion of bacterial lipopolysaccharide J Neurochem 2004 88 1168 1178 10.1046/j.1471-4159.2003.02246.x 15009672

[B13] Lin TN Wang Q Simonyi A Chen JJ Cheung WM He YY Xu J Sun AY Hsu CY Sun GY Induction of secretory phospholipase A2 in reactive astrocytes in response to transient focal cerebral ischemia in the rat brain J Neurochem 2004 90 637 645 10.1111/j.1471-4159.2004.02540.x 15255941

[B14] Pinto F Brenner T Dan P Krimsky M Yedgar S Extracellular phospholipase A2 inhibitors suppress central nervous system inflammation Glia 2003 44 275 282 10.1002/glia.10296 14603468

[B15] Wang G Daniel BM DeCoster MA Role of nitric oxide in regulating secreted phospholipase A2 release from astrocytes Neuroreport 2005 16 1345 1350 10.1097/01.wnr.0000174403.79020.65 16056137

[B16] Schwemmer M Aho H Michel JB Interleukin-1beta-induced type IIA secreted phospholipase A2 gene expression and extracellular activity in rat vascular endothelial cells Tissue Cell 2001 33 233 240 10.1054/tice.2000.0163 11469536

[B17] Rupprecht G Scholz K Beck KF Geiger H Pfeilschifter J Kaszkin M Cross-talk between group IIA-phospholipase A2 and inducible NO-synthase in rat renal mesangial cells Br J Pharmacol 1999 127 51 56 10.1038/sj.bjp.0702500 10369455 PMC1565987

[B18] Huang H Rose JL Hoyt DG p38 Mitogen-activated protein kinase mediates synergistic induction of inducible nitric-oxide synthase by lipopolysaccharide and interferon-gamma through signal transducer and activator of transcription 1 Ser727 phosphorylation in murine aortic endothelial cells Mol Pharmacol 2004 66 302 311 10.1124/mol.66.2.302 15266021

[B19] Wu F Tyml K Wilson JX iNOS expression requires NADPH oxidase-dependent redox signaling in microvascular endothelial cells J Cell Physiol 2008 217 207 214 10.1002/jcp.21495 18481258 PMC2551742

[B20] Badamtseren B Odkhuu E Koide N Haque A Naiki Y Hashimoto S Komatsu T Yoshida T Yokochi T Thalidomide inhibits interferon-γ-mediated nitric oxide production in mouse vascular endothelial cells Cell Immunol 2011 270 19 24 10.1016/j.cellimm.2011.03.018 21477797

[B21] Abbott NJ Hughes CCW Revest PA Greenwood J Development and characterisation of a rat brain capillary endothelial culture: towards an in vitro blood–brain barrier J Cell Sci 1992 103 23 37 1429907 10.1242/jcs.103.1.23

[B22] Mounier CM Ghomashchi F Lindsay MR James S Singer AG Parton RG Gelb MH Arachidonic acid release from mammalian cells transfected with human groups IIA and X secreted phospholipase a (2) occurs predominantly during the secretory process and with the involvement of cytosolic phospholipase A(2)-alpha J Biol Chem 2004 279 25024 25038 10.1074/jbc.M313019200 15007070

[B23] Liu T Li Y Lin K Yin H He B Zheng M Wang G Regulation of S100A4expression via the JAK2-STAT3 pathway in rhomboid-phenotype pulmonary arterial smooth muscle cells exposure to hypoxia Int J Biochem Cell Biol 2012 44 1337 1345 10.1016/j.biocel.2012.04.017 22561747

[B24] Li Y Wang G Lin K Yin H Zhou C Liu T Wu G Qian G Rab1 GTPase promotes expression of beta-adrenergic receptors in rat pulmonary microvascular endothelial cells Int J Biochem Cell Biol 2010 42 1201 1209 10.1016/j.biocel.2010.04.009 20417717 PMC4792279

[B25] Chang YS Munn LL Hillsley MV Dull RO Yuan J Lakshminarayanan S Effect of vascular endothelial growth factor on cultured endothelial cell monolayer transport properties Microvasc Res 2000 59 265 277 10.1006/mvre.1999.2225 10684732

[B26] Yagami T Ueda K Sakaeda T Okamura N Nakazato H Kuroda T Hata S Sakaguchi G Itoh N Hashimoto Y Fujimoto M Effects of an endothelin B receptor agonist on secretory phospholipase A2-IIA-induced apoptosis in cortical neurons Neuropharmacology 2005 48 291 300 10.1016/j.neuropharm.2004.09.011 15695168

[B27] Chiricozzi E Fernandez-Fernandez S Nardicchi V Almeida A Bolaños JP Goracci G Group IIA secretary phospholipase A2 (GIIA) mediates apoptotic death during NMDA receptor activation in rat primary cortical neurons J Neurochem 2010 112 1574 1583 10.1111/j.1471-4159.2010.06567.x 20067579

[B28] Yagami T Ueda K Asakura K Hata S Kuroda T Sakaeda T Takasu N Tanaka K Gemba T Hori Y Human group IIA secretory phospholipase A2 induces neuronal cell death via apoptosis Mol Pharmacol 2002 61 114 126 10.1124/mol.61.1.114 11752212

[B29] Yagami T Ueda K Asakura K Hayasaki-Kajiwara Y Nakazato H Sakaeda T Hata S Kuroda T Takasu N Hori Y Human group IIA secretory phospholipase A2 potentiates Ca2+ influx through L-type voltage-sensitive Ca2+ channels in cultured rat cortical neurons J Neurochem 2003 85 749 758 10.1046/j.1471-4159.2003.01712.x 12694401

[B30] Lin TN Wang Q Simonyi A Chen JJ Cheung WM He YY Xu J Sun AY Hsu CY Sun GY Induction of secretory phospholipase A2 in reactive astrocytes in response to transient focal cerebral ischemia in the rat brain J Neurochem 2004 90 637 645 10.1111/j.1471-4159.2004.02540.x 15255941

[B31] Jensen MD Sheng W Simonyi A Johnson GS Sun AY Sun GY Involvement of oxidative pathways in cytokine-induced secretory phospholipase A2-IIA in astrocytes Neurochem Int 2009 55 362 368 10.1016/j.neuint.2009.04.002 19375465 PMC2768481

[B32] Park DW Kim JR Kim SY Sonn JK Bang OS Kang SS Kim JH Baek SH Akt as a mediator of secretory phospholipase A2 receptor-involved inducible nitric oxide synthase expression J Immunol 2003 170 2093 2099 12574380 10.4049/jimmunol.170.4.2093

[B33] Baek SH Kwon TK Lim JH Lee YJ Chang HW Lee SJ Kim JH Kwun KB Secretory phospholipase A2-potentiated inducible nitric oxide synthase expression by macrophages requires NF-kappa B activation J Immunol 2000 164 6359 6365 10843690 10.4049/jimmunol.164.12.6359

[B34] Li W Xia J Sun GY Cytokine induction of iNOS and sPLA2 in immortalized astrocytes (DITNC): response to genistein and pyrrolidine dithiocarbamate J Interferon Cytokine Res 1999 19 121 127 10.1089/107999099314261 10090397

[B35] Wang JH Sun GY Ethanol inhibits cytokine-induced iNOS and sPLA2 in immortalized astrocytes: evidence for posttranscriptional site of ethanol action J Biomed Sci 2001 8 126 133 11173986 10.1007/BF02255981

[B36] Lundblad C Grände PO Bentzer P Hemodynamic and histological effects of traumatic brain injury in eNOS-deficient mice J Neurotrauma 2009 26 1953 1962 10.1089/neu.2009.0955 19929218

[B37] Wu KK Regulation of endothelial nitric oxide synthase activity and gene expression Ann N Y Acad Sci 2002 962 122 130 10.1111/j.1749-6632.2002.tb04062.x 12076969

[B38] Huang H Rose JL Hoyt DG Mitogen-activated protein kinase mediates synergistic induction of inducible nitric-oxide synthase by lipopolysaccharide and interferon-gamma through signal transducer and activator of transcription 1 Ser727 phosphorylation in murine aortic endothelial cells Mol Pharmacol 2004 66 302 311 10.1124/mol.66.2.302 15266021

[B39] Huang A Zhang YY Chen K Hatakeyama K Keaney JF Cytokine-stimulated GTP cyclohydrolase I expression in endothelial cells requires coordinated activation of nuclear factor-kappaB and Stat1/Stat3 Circ Res 2005 96 164 171 10.1161/01.RES.0000153669.24827.DF 15604419

[B40] Wu F Han M Wilson JX Tripterine prevented endothelial barrier dysfunction by inhibiting endogenous peroxynitrite formation Br J Pharmacol 2009 157 1014 1023 10.1111/j.1476-5381.2009.00292.x 19508391 PMC2737660

[B41] Saura M Zaragoza C Bao C Herranz B Rodriguez-Puyol M Lowenstein C Stat3 mediates interleukin-6 [correction of interelukin-6] inhibition of human endothelial nitric-oxide synthase expression J Biol Chem 2006 281 30057 30062 10.1074/jbc.M606279200 16887796

[B42] Elsasser TH Li CJ Caperna TJ Kahl S Schmidt WF Growth hormone (GH)-associated nitration of Janus kinase-2 at the 1007Y-1008Y epitope impedes phosphorylation at this site: mechanism for and impact of a GH, AKT, and nitric oxide synthase axis on GH signal transduction Endocrinology 2007 148 3792 3802 10.1210/en.2006-1736 17510232

[B43] Lee ST Chu K Sinn DI Jung KH Kim EH Kim SJ Kim JM Ko SY Kim M Roh JK Erythropoietin reduces perihematomal inflammation and cell death with eNOS and STAT3 activations in experimental intracerebral hemorrhage J Neurochem 2006 96 1728 1739 10.1111/j.1471-4159.2006.03697.x 16539688

[B44] Gu Y Zheng G Xu M Li Y Chen X Zhu W Tong Y Chung SK Liu KJ Shen J Caveolin-1 regulates nitric oxide-mediated matrix metalloproteinases activity and blood–brain barrier permeability in focal cerebral ischemia and reperfusion injury J Neurochem 2012 120 147 156 10.1111/j.1471-4159.2011.07542.x 22007835

[B45] Brochu ME Girard S Lavoie K Sébire G Developmental regulation of the neuroinflammatory responses to LPS and/or hypoxia-ischemia between preterm and term neonates: An experimental study J Neuroinflammation 2011 8 55 10.1186/1742-2094-8-55 21599903 PMC3121616

[B46] Trousson A Makoukji J Petit PX Bernard S Slomianny C Schumacher M Massaad C Cross-talk between oxysterols and glucocorticoids: differential regulation of secreted phopholipase A2 and impact on oligodendrocyte death PLoS One 2009 4 e8080 10.1371/journal.pone.0008080 19956653 PMC2779104

[B47] Menschikowski M Hagelgans A Siegert G Secretory phospholipase A2 of group IIA: is it an offensive or a defensive player during atherosclerosis and other inflammatory diseases? Prostaglandins Other Lipid Mediat 2006 79 1 33 10.1016/j.prostaglandins.2005.10.005 16516807

[B48] Ravaux L Denoyelle C Monne C Limon I Raymondjean M El-Hadri K Inhibition of interleukin-1beta-induced group IIA secretory phospholipase A2 expression by peroxisome proliferator-activated receptors (PPARs) in rat vascular smooth muscle cells: cooperation between PPARbeta and the proto-oncogene BCL-6 Mol Cell Biol 2007 27 8374 87 10.1128/MCB.00623-07 17908795 PMC2169168

